# Advanced Green Materials: Sustainable Cellulose–Lignin Composite Films Prepared via Ionic Liquid Processing

**DOI:** 10.3390/polym18020211

**Published:** 2026-01-13

**Authors:** Witold Madaj, Michał Puchalski, Konrad Sulak, Dariusz Wawro, Ewelina Pabjańczyk-Wlazło

**Affiliations:** 1 Textile Institute, Lodz University of Technology, 116 Zeromskiego Street, 90-543 Lodz, Poland; witold.madaj@dokt.p.lodz.pl (W.M.); ewelina.pabjanczyk-wlazlo@p.lodz.pl (E.P.-W.); 2 Biopolymers and Chemical Fibers Center, Łukasiewicz Research Network-Lodz Institute of Technology, 19/27 Sklodowskiej-Curie Street, 90-570 Lodz, Poland; konrad.sulak@lit.lokasiewicz.gov.pl (K.S.); dariusz.wawro@lit.lkasiewicz.gov.pl (D.W.)

**Keywords:** ionic liquid, cellulose, lignin, film, composite

## Abstract

The article presents the preparation method of a green composite material composed of cellulose and lignin using an ionic liquid as a solvent. In the process, cellulose and lignin are dissolved in the ionic liquid and subsequently regenerated into a composite film via coagulation in ethanol/water bath. The research focused on evaluating the mechanical properties of the resulting composite, which exhibited a high tensile strength exceeding 100 MPa, demonstrating its robustness and potential for various applications. Importantly, the simultaneous integration of lignin enabled a favorable balance between high mechanical strength and enhanced biodegradability, addressing a common trade-off in sustainable materials. Additionally, the biodegradation behavior of the composite in soil was investigated, showing that it gradually decomposes, making it environmentally friendly. Toxicity tests on soil bacteria indicated that the composite does not adversely affect microbial activity, supporting its suitability for ecological use. Furthermore, the gas permeability and water vapor transmission of the composite film was assessed, providing insight into its barrier properties. Overall, the study highlights the potential of cellulose-lignin composites produced via ionic liquids as sustainable and biodegradable materials with promising mechanical and environmental properties.

## 1. Introduction

The growing awareness of the gradual yet inevitable depletion of fossil fuel resources—and, consequently, of the raw materials required for synthetic polymer production—has driven the demand for durable and environmentally sustainable materials. The foundation for developing such materials lies in renewable, biodegradable, and easily recyclable raw resources. The advancement of eco-friendly composite materials incorporating natural fibers such as cotton, flax, and hemp offers a viable alternative to conventional synthetic fibers. Furthermore, green composites utilize matrix materials derived from biopolymers or resins obtained from starch, cellulose, or proteins. These green composites represent a new generation of high-performance materials that integrate natural fibers within a polymer matrix to produce lightweight, strong, recyclable, and biodegradable composites. The use of renewable resources, including plants and microorganisms, in the fabrication of green composites significantly reduces dependence on petrochemical products, thereby mitigating the depletion of natural resources on a global scale [1,2,3,4,5].

Despite the considerable advantages of green composites, certain challenges persist regarding their practical application. Natural fibers exhibit lower homogeneity compared to glass or carbon fibers, tend to absorb moisture, possess inferior mechanical properties, and present difficulties in bonding with the composite matrix. Consequently, they require pretreatment prior to incorporation. In addition, the development of suitable matrices or binders for natural fiber reinforcement remains relatively underexplored and demands further research. The primary issue lies in poor adhesion and weak interfacial bonding between natural fibers and the matrix [6,7,8,9,10].

The diversity of ionic liquids (ILs) and their broad spectrum of interactions—particularly with organic substances—renders them highly suitable for green composite production. Ionic liquids exhibit unique properties, including negligible vapor pressure, low melting points, non-flammability, facile regeneration, and thermal stability across a wide temperature range, making them excellent solvents for organic compounds without posing significant hazards during processing. Moreover, many ionic liquids are environmentally benign and biodegradable. Their anions strongly influence hydrogen bonding within molecular structures, facilitating the disruption of cellulose and hemicellulose networks and, under prolonged and intensive treatment, even of lignin. Owing to these characteristics, ionic liquids can effectively dissolve both lignin and cellulose [11,12,13].

The resulting cellulose–lignin composites can be compared to cellulose films produced using ionic liquids. Cellulose films obtained via the viscose process have a wide range of applications, primarily as food packaging materials. Cellophane, a well-established cellulose-based film, is widely used for this purpose due to its non-toxic nature. However, its industrial production involves toxic and environmentally harmful chemicals, prompting the search for alternative, safer, and more sustainable manufacturing methods. One promising approach involves the use of imidazolium-based ionic liquids as cellulose solvents for film formation [14,15,16].

Hameed et al. [17] demonstrated that ionic liquids, specifically 1-butyl-3-methylimidazolium chloride ([bmim]Cl), can be employed to fabricate wool–cellulose composite films. These films, coagulated in demineralized water, exhibited moderate tensile strength of 54 MPa. Similarly, Takegawa et al. [18] investigated a mixture of [amim]Br and [bmim]Cl for producing chitin–cellulose composite films using microcrystalline cellulose (Avicel) as the matrix. However, these films displayed relatively weak mechanical properties, with tensile strength of 10 MPa and elongation of 10%. Likewise, chitosan–cellulose composite films formed from [bmim][OAc] exhibited comparable performance. In contrast, incorporating poly(vinyl alcohol) (PVA) into a solution of cotton linters in 1-allyl-3-methylimidazolium chloride ([amim]Cl) significantly enhanced the tensile strength of the resulting cellulose films. After coagulation in water, transparent films were obtained with tensile strength of 100 MPa and elongation of 25%. The highest tensile strength values, reaching up to 103 MPa with elongation of 30%, were achieved by casting cellulose solutions in 1-ethyl-3-methylimidazolium acetate ([emim][OAc]) [17,18,19,20,21].

This study introduces an environmentally friendly method for producing cellulose–lignin composite films using ionic liquids as solvents. The process involves dissolving both biopolymers in an ionic liquid and regenerating them into films through coagulation in an ethanol–water bath. The research examines key functional properties of the composite, including mechanical strength, biodegradability, ecotoxicity, and barrier performance. Results indicate that the material combines robustness with environmental compatibility, showing no adverse effects on soil microorganisms and exhibiting favorable barrier characteristics. These findings highlight the potential of cellulose–lignin composites prepared via ionic liquids as sustainable alternatives to conventional materials.

## 2. Materials and Methods

### 2.1. Materials

Softwood kraft dissolving pulp (V67) from Buckeye Technologies (Memphis, TN, USA), was utilized in this study, with a viscosity of 408 mL/g, and containing 92.5% R10 and 97.1% R18 fractions. The ionic liquid 1-ethyl-3-methylimidazolium acetate ([emim][OAc]), with a purity of 95%, was sourced from Merck KGaA (Darmstadt, Germany). Lignin, (lignin kraft) with a purity of 95%, a sintering temperature of 188 °C, and a pH of 6.5 (in a 5% aqueous solution), was obtained from Sigma-Aldrich (Burlington, MA, USA). Additionally, ethyl alcohol was provided by Linegal Chemicals Company (Warsaw, Poland).

### 2.2. Preparation of Cellulose/Lignin/IL Solution

In order to get rid of water, cellulose and lignin were separately dried at 90 °C in a vacuum dryer at a pressure of 0.1 bar before being separately dissolved in ionic liquids. Then, separate solutions were prepared from the dried cellulose/lignin and ionic liquid. The resulting mixtures were thoroughly mixed using an ultrasonic homogenizer for 15 min and then placed in a vacuum oven. Mixtures were heated until a clear solution was obtained. The solutions of individual ionic liquids, cellulose and lignin were combined and mixed thoroughly with an ultrasonic homogenizer for 15 min. Then the resulting ionic liquid-cellulose-lignin solution was heated in a dryer until it was completely de-aerated.

### 2.3. Casting of the Cellulose/Lignin Film Composite

The cellulose/lignin/[emim][OAc] solution was poured onto smooth sheets of metal and carefully spread to obtain an even layer of the solution on the surface of the sheet. The mixture was regenerated using a regeneration bath, 80% ethyl alcohol/20% water. Regeneration was carried out until the resulting composite was completely coagulated. Then the resulting films were rinsed in water for 24 h. After the first hour of rinsing, the water was changed. After rinsing, the foils were dried. The drying process was carried out for 24 h at a temperature of 23 ± 1 °C and a humidity of 20% or 50%.

### 2.4. Mechanical Properties of the Composites

The mechanical properties of the foil composite were measured using an Instron 5544 tensile force measuring device (Norwood, MA, USA), in accordance with the PN-ISO 4593:1999 and PN-EN ISO 527-3:2019-01 standards [22,23].

### 2.5. Determination of the Degree of Composite Decomposition in the Soil Environment

Tests of the composite were conducted according to—“Determination of the degree of decomposition of plastics and textile products in simulated soil conditions on a laboratory scale. Determination of mass loss” (PN-EN ISO 11 266:2020-11, PN-EN ISO 11 721-1:2002, PN-EN ISO 11 721-2:2005) [24,25,26]. Formula for percentage weight loss:

(1) $$Weight\;loss\left[\%\right]=100-(\frac{m_{f}}{m_{i}}\times 100)$$

$m_{f}$—Final mass of the sample

$m_{i}$—Initial mass of the sample

### 2.6. The Impact of the Composite on the Growth of Soil Microorganisms—Ecotoxicity Test

The research was conducted in accordance with established standards for the assessment of the impact of materials of natural and synthetic origin on soil microflora. Specifically, microbiological analyses were performed following the PN-EN ISO 4833-1:2013-12 standard [27], “Microbiology of the food chain—Horizontal method for determining the number of microorganisms by deep inoculation at 30 °C.” General requirements and principles for microbiological testing were applied in accordance with the PN-EN ISO 7218:2024-12 standard [28], “Microbiology of food and feed—General requirements and principles of microbiological tests.” In addition, the estimation of measurement uncertainty was carried out following the guidelines of PN-EN ISO 19036:2020-04 for quantitative methods [29]. Based on these standardized procedures, the impact of the tested samples on soil microflora was assessed using the deep plate method to determine the total number of microorganisms.

### 2.7. Gas Permeability Through Foil

The PN-EN ISO 2556:2002 norm was used, to determine gas permeability in plastics using the manometric method, a pre-conditioned foil sample (23 °C, 50% humidity) is placed in a gas diffusion chamber, separating two test chambers [30]. After removing air from both chambers, test gas is introduced into the higher-pressure chamber, creating a pressure difference. The amount of gas that passes through the sample is measured by monitoring the pressure increase in the lower chamber.

### 2.8. Transmission of Water Vapor Through the Foil

Water vapor permeability is the amount of vapor, expressed in grams, that diffuses through the surface of 1 m^2^ of the tested foil during 1 day, at 85% humidity, at a temperature of 20 °C. Water vapor transmission through the foils was tested in accordance with the American standard ASTM E96 “Standard Test Methods for Water Vapor Transmission of Materials” [31].

## 3. Results

### 3.1. Mechanical Properties

**Figure 1:**
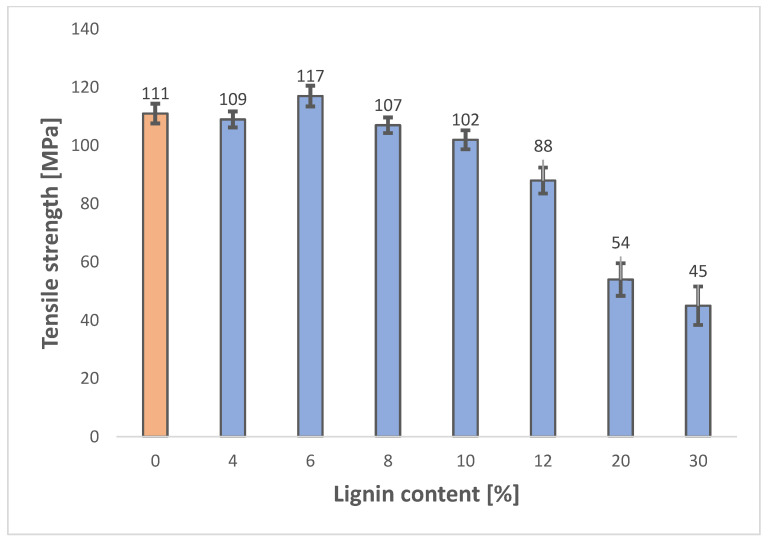
Tensile strength of cellulose/lignin composite films as a function of lignin content.

The cellulose/lignin/ionic liquid (IL) solutions were formulated to achieve a dry matter content of 10%. The resulting composites exhibit notable mechanical strength. The reference sample composed solely of cellulose demonstrated a tensile strength of 111 MPa, which is consistent with values reported in previous studies by Wawro et al. [21]. The highest tensile strength was obtained for the composite containing 6% lignin (Table 1). Incorporating lignin up to 10% maintained tensile strength above 100 MPa. However, lignin contents exceeding 10% significantly reduced the mechanical performance of the composites. Specifically, at 20% and 30% lignin, tensile strengths decreased to 54 MPa and 45 MPa (Figure 1), respectively—representing approximately a 50% reduction compared to the optimal composition. For lignin contents of 40% and 50%, continuous composite films could not be formed; during drying, surface integrity was compromised due to stress-induced cracking.

### 3.2. Morphological Analyzes

**Figure 2:**
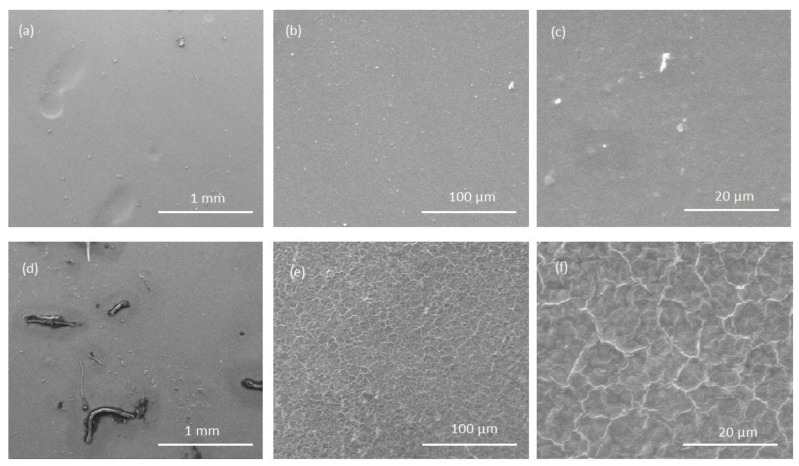
The SEM images comparison of the surfaces of cellulose films and cellulose/lignin composite films. Images (**a**–**c**) show of pure cellulose film. Images (**d**–**f**) show cellulose/lignin composite film.

Surface morphology of cellulose films and cellulose/lignin composite films was examined using scanning electron microscopy (SEM) with a Quanta 200 (W) system (FEI Co., Eindhoven, The Netherlands). Images (a), (b) and (c) show of pure cellulose film. Images (d), (e) and (f) show cellulose/lignin composite film. At a magnification of 100×, both the pure cellulose film and the cellulose–lignin composite exhibited a relatively homogeneous and smooth surface. However, at higher magnifications (1000× and 5000×), distinct differences became apparent. The composite film displayed a less uniform surface with visible textural features, indicating the presence of lignin domains within the matrix (Figure 2). Despite these morphological variations, no adverse effect on the tensile strength of the films was observed.

### 3.3. Decomposition in the Soil Environment

Cellulose films were prepared from a 10% cellulose solution in an ionic liquid (IL), while cellulose/lignin composite films were obtained from a 10% dry matter solution in IL, consisting of 90% cellulose and 10% lignin. Biodegradation tests were conducted on samples in three forms: (i) dried films, (ii) wet films not subjected to drying, and (iii) unrinsed films removed directly from the coagulation bath after formation. After the first week of soil exposure, a pronounced difference in decomposition was observed between the reference cellulose film and the cellulose/lignin composite. The composite containing 10% lignin exhibited a mass loss of 24.9%, whereas the pure cellulose film degraded by only 3.97% (Table 1).

**Figure 3:**
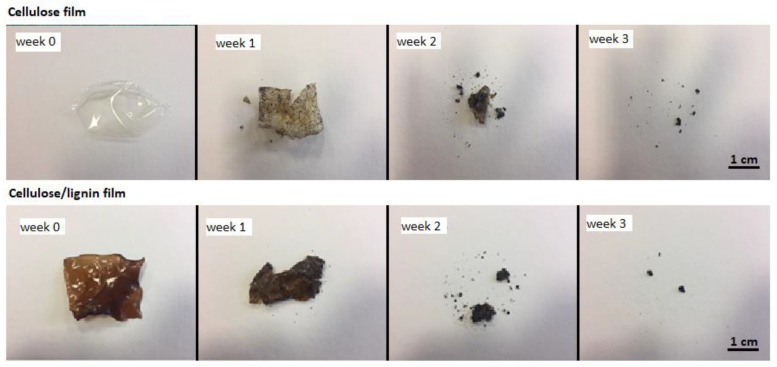
The visual comparison of the weight loss of the cellulose foil and the composite after successive weeks of biodegradation.

In subsequent weeks, these differences diminished. By week 2, both film types showed approximately 50% mass loss, and by week 3, nearly complete degradation was achieved. Wet and unrinsed samples degraded significantly faster, losing about 80% of their mass within the first week and reaching almost complete decomposition by week 2. This accelerated biodegradation is attributed to the high water content of these samples. Importantly, residual ionic liquid did not inhibit the biodegradation process. A visual representation of the progressive weight loss for cellulose and composite films is provided in Figure 3.

### 3.4. The Impact of the Composite on the Growth of Soil Microorganisms

An ecotoxicity assessment was performed to evaluate the impact of cellulose and cellulose/lignin composite films on soil microbial growth. The study was based on quantifying the number of microbial cells in soil samples containing the tested films compared to a control soil sample. Experiments were conducted under controlled conditions (temperature: 30 °C; humidity: 70%). The initial microbial population in the soil was approximately 10^7^ cfu/g. Changes in microbial counts over time for different film types are presented in Table 2.

After 14 and 21 days, a marked decrease in microbial numbers was observed across all samples, which can be attributed to environmental changes caused by the introduction of the films. This alteration likely increased competition among microorganisms, favoring those capable of utilizing film components as a nutrient source. Consequently, the initial decline in microbial counts reflects displacement of non-adapted species. By day 28, microbial numbers began to rise again, indicating that adapted microorganisms had proliferated after outcompeting others. Importantly, no signs of toxicity were detected in any sample, confirming that the films did not inhibit microbial growth.

### 3.5. Gas Permeability and Transmission of Water Vapor

Cellulose films were prepared from a 10% cellulose solution in an ionic liquid (IL), while cellulose/lignin composite films were obtained from a 10% dry matter solution in IL, consisting of 90% cellulose and 10% lignin. The resulting films exhibited high barrier properties, with cellulose and composite films showing comparable performance. The incorporation of lignin did not significantly affect gas permeability or water vapor transmission. Oxygen and carbon dioxide permeability values remained below 2.3 cm^3^/m^2^·24 h·0.1 MPa, which is substantially lower than those of commonly used plastic films such as polycarbonate (~498 cm^3^/m^2^·24 h·0.1 MPa), polyethylene (~344 cm^3^/m^2^·24 h·0.1 MPa), or polyethylene glycol terephthalate (~6 cm^3^/m^2^·24 h·0.1 MPa) [32], known for its low permeability (Table 3). However, the obtained films were not fully homogeneous. In some permeability tests, samples exhibited unrestricted gas flow due to structural imperfections such as holes, air pockets, or cracks formed during the manufacturing process. These defects compromised barrier integrity and explain the variability observed in certain measurements.

## 4. Discussion

The findings of this study confirm the strong influence of lignin content on the mechanical performance of cellulose-based composites. While moderate lignin incorporation (around 6%) enhanced tensile strength to 117 MPa, excessive lignin (>12%) led to a marked decline in strength, consistent with recent reports that attribute this effect to disrupted hydrogen bonding and phase separation within the polymer matrix [33]. These observations align with the growing consensus that lignin acts as a reinforcing agent only within an optimal concentration range, beyond which its rigid, amorphous structure compromises film cohesion and flexibility.

The morphological analysis revealed surface irregularities at high magnifications, which likely contributed to the variability in barrier performance. Although sections of the film exhibited excellent oxygen and carbon dioxide permeability values—comparable to advanced cellulose nanofiber-based coatings [34]—the presence of cavities and cracks resulted in localized permeability failures. This underscores the importance of refining processing conditions, particularly coagulation and drying steps, to achieve uniform film morphology. Recent studies have demonstrated that chemical crosslinking and nanofiller incorporation can significantly improve barrier properties under humid conditions, suggesting potential strategies for future optimization [35].

Biodegradation tests confirmed rapid decomposition of both cellulose and cellulose–lignin films, with nearly complete degradation within three weeks. This behavior is consistent with recent findings that lignin incorporation can modulate biodegradation rates, slowing microbial attack at higher concentrations while maintaining overall compostability [36,37]. Importantly, the absence of ecotoxic effects on soil microorganisms supports the environmental safety of ionic liquid-based processing, echoing previous reports that emphasize the low toxicity and recyclability of imidazolium-based solvents when properly recovered [38,39].

From an application perspective, the combination of high tensile strength at optimal lignin content, rapid biodegradability, and non-toxic behavior positions these composites as promising candidates for sustainable packaging and agricultural uses. However, the variability in barrier performance highlights a critical challenge for commercialization. Advanced approaches such as multilayer structures or surface functionalization—already explored in nanocellulose-based films—could be adapted to cellulose–lignin systems to enhance moisture resistance and gas impermeability [40]. Furthermore, the demonstrated recyclability of ionic liquids in similar systems suggests that scaling up these processes could align with circular economy principles, reducing environmental impact while maintaining material performance [41].

Overall, this study contributes to the growing body of evidence that cellulose–lignin composites processed in ionic liquids represent a sustainable alternative to petroleum-based plastics. Future research should focus on optimizing lignin content, improving film homogeneity, and integrating functionalization strategies to tailor barrier properties for specific applications.

## 5. Conclusions

Cellulose–lignin composite films were successfully fabricated by dissolving both biopolymers in the ionic liquid 1-ethyl-3-methylimidazolium acetate ([Emim][OAc]) and regenerating the solution into continuous films. The study demonstrates that lignin incorporation exerts a significant influence on the mechanical and functional properties of the composite. An optimal lignin concentration of approximately 6% yielded the highest tensile strength (117 MPa), confirming the reinforcing effect of lignin at moderate levels. However, increasing lignin content beyond 12% resulted in a pronounced decline in mechanical performance, with reductions of up to 50% at 20–30% lignin. At concentrations above 40%, film formation was not feasible, indicating a critical threshold for processability and structural integrity.

Although the surface of the composite appeared macroscopically homogeneous, barrier property tests revealed localized imperfections such as cavities and cracks, which compromised gas impermeability. Defect-free regions exhibited excellent barrier performance, with oxygen and carbon dioxide permeability values (1.62 and 2.21 cm^3^/m^2^, 24 h, 0.1 MPa, respectively) significantly lower than those of conventional plastics. These findings highlight the potential for optimizing processing conditions—particularly coagulation and drying steps—to improve film uniformity and ensure consistent barrier properties.

Environmental assessments confirmed the strong sustainability profile of the material. Biodegradation tests demonstrated complete decomposition within three weeks under soil conditions, and ecotoxicity studies indicated no inhibitory effects on soil microbial activity, even in the presence of residual ionic liquid. These results validate the ecological compatibility of both the composite and the processing solvent, supporting their potential integration into sustainable material systems.

Overall, cellulose–lignin composites produced via ionic liquid processing represent a promising class of bio-based materials combining mechanical robustness, biodegradability, and environmental safety. Further research should focus on refining processing techniques to eliminate structural defects and tailoring material properties for specific applications such as packaging and agricultural films.

## Figures and Tables

**Table 1:** The loss of composite mass during decomposition in the soil environment for composite cellulose/lignin ratio 90%/10%.

Sample	Weight Loss (%)		
	Week 1	Week 2	Week 3
Cellulose film	3.97	44.9	96.8
Wet Cellulose film	83.4	98.7	98.8
Cellulose/lignin composite film	24.9	50.8	97.9
Wet Cellulose/lignin composite film	84.3	99.6	99.9

**Table 2:** The change in the number of microorganisms (cfu/g) in the soil containing film samples over time.

Sample	Number of Microorganisms (cfu/g)			
	7 Days	14 Days	21 Days	28 Days
Cellulose film	2.1 × 10^8^	2.0 × 10^7^	1.3 × 10^6^	4.3 × 10^6^
Wet Cellulose film	3.9 × 10^7^	1.0 × 10^6^	4.1 × 10^6^	6.9 × 10^6^
Cellulose/lignin composite film	8.0 × 10^7^	2.0 × 10^6^	1.4 × 10^6^	1.6 × 10^6^
Wet Cellulose/lignin composite film	3.7 × 10^7^	5.0 × 10^6^	8.7 × 10^5^	4.7 × 10^6^

**Table 3:** The comparison of the barrier properties of the composite film with the reference cellulose film.

Sample	Permeability O_2_	Permeability CO_2_	Water Vapor Transmission
	(cm^3^/m^2^·24 h·0.1 MPa) 23 °C, 50% RH	(cm^3^/m^2^·24 h·0.1 MPa) 23 °C, 50% RH	(g/m^2^·h) 23 °C, 24 h
Cellulose film	1.41	1.97	45.3
Cellulose/lignin composite film	1.62	2.21	50.3

## Data Availability

The raw data supporting the conclusions of this article will be made available by the authors on request.

## Abbreviations

The following abbreviations are used in this manuscript:

IL	Ionic Liquids
[bmim]Cl	1-butyl-3-methylimidazolium chloride
[amim]Cl	1-allyl-3-methylimidazolium chloride
[emim][OAc]	1-ethyl-3-methylimidazolium acetate
SEM	Scanning Electron Microscopy
PVA	poly(vinyl alcohol)
